# Flow-Induced Protein Chain Deformation, Segmental Orientation, and Phase Separation in Native Silk Feedstock

**DOI:** 10.1021/acs.biomac.3c00233

**Published:** 2023-05-26

**Authors:** Peter R. Laity, Gary Dunderdale, Oleksandr O. Mykhaylyk, Chris Holland

**Affiliations:** † Department of Materials Science and Engineering, University of Sheffield, Sir Robert Hadfield Building, Mappin Street, Sheffield S1 3JD, U.K.; ‡ Sustainable Aviation Fuels Innovation Centre, University of Sheffield, Sheffield Business Park, Europa Avenue, Sheffield S9 1ZA, U.K.; § Department of Chemistry, University of Sheffield, Dainton Building, Brook Hill, Sheffield S3 7HF, U.K.

## Abstract

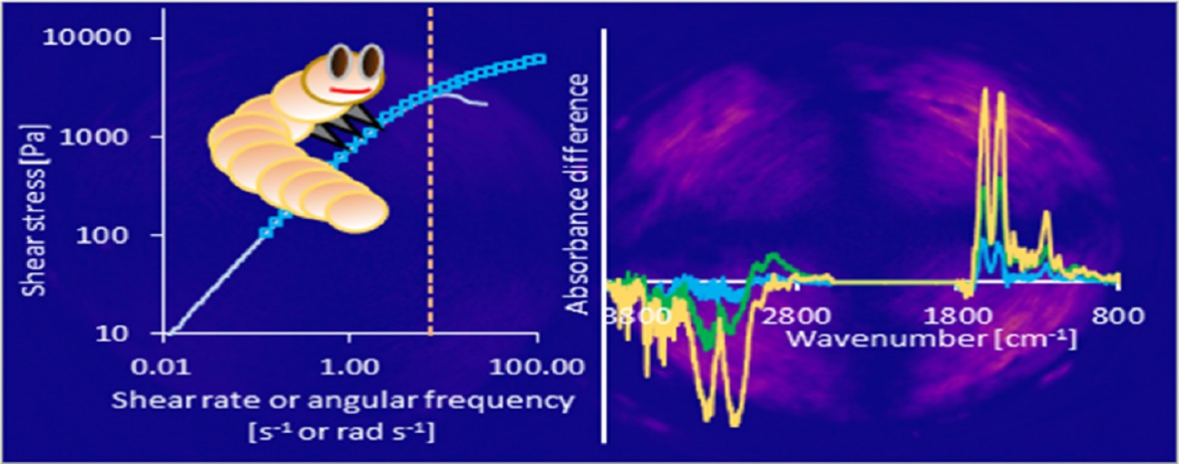

The ability of many arthropods to spin silk and its many uses bear testament to its importance in Nature. Despite over a century of research, however, the spinning process is still not fully understood. While it is widely accepted that flow and chain alignment may be involved, the link to protein gelation remains obscure. Using combinations of rheology, polarized light imaging, and infrared spectroscopy to probe different length scales, this work explored flow-induced gelation of native silk feedstock from *Bombyx mori* larvae. Protein chain deformation, orientation, and microphase separation were observed, culminating in the formation of antiparallel β-sheet structures while the work rate during flow appeared as an important criterion. Moreover, infrared spectroscopy provided direct observations suggesting a loss of protein hydration during flow-induced gelation of fibroin in native silk feedstock, which is consistent with recently reported hypotheses.

## Introduction

The ability to spin silk is widely found in insects, myriapods, and spiders,^1−6^ with the fibers serving a multitude of purposes. Examples include cocoon construction or structural elements in the nests of bees;^7^ the production of protective webs and cocoons by lepidopteran larvae (caterpillars) prior to pupation;^8−10^ sperm webs produced by centipedes,^11,12^ silverfish,^13^ and some spiders;^14,15^ and wrapping of “nuptial gifts” offered by some male spiders to distract the female during mating.^16^ Indeed, spiders themselves provide many diverse examples of silk use, such as webs for capturing prey, the linings of burrows, “diving bells” of aquatic species, and windblown threads for aerial dispersal, allowing spiders to successfully exploit many different ecological niches.^17−21^

Despite the ubiquity of silk production by many arthropods and over a century of research, the mechanisms by which this occurs remain at least partially obscured. It has been known for many years that the fibers are produced from an aqueous protein solution under ambient conditions, with the phase change from the solution to solid state being initiated by flow.^22−25^ In natural fiber spinning, changes in pH and salinity within the silk duct may also be important,^26−33^ affecting protein oligomerization through interactions between terminal domains. Recent rheological studies^34−36^ have shown that the gelation of fibroin in native silk feedstock (NSF) from *Bombyx mori* silkworms can be initiated by flow alone, however, without the need for compositional changes. Moreover, once initiated, the gel can develop further (i.e., becoming stiffer) under quiescent conditions, without further flow stimulation.^35^

Various studies have suggested the importance of a critical shear rate, shear stress, or energy accumulation to initiate gelation.^22−25,34−36^ It is well known that flow can produce chain deformation and orientation in polymer solutions^37−48^ and silk fibers are highly oriented at the molecular, crystalline, and fibrillar levels;^49−56^ hence, it seems likely that some aspect of flow-induced chain deformation and orientation may underpin the gelation process. Based on the hypothesis of an “entropically penalized hydration shell” enslaved to the fibroin, it was suggested previously^35^ that flow-induced gelation may occur through desolvation due to chain deformation causing a further loss of entropy of the bound water. Standard molecular theories of polymer elasticity describe the chains as “entropic springs”, for which stretching restricts the number of accessible conformations and causes an increase in free energy through the corresponding loss of entropy.^57−61^ Consequently, this explanation of flow-induced gelation assumes that the accessibility of chain conformations also affects the entropy of the associated hydration shell. In this respect, it is interesting that several researchers^39,62,63^ have reported correlations between the orientation of polymer chain segments and the associated solvent, suggesting that any constraints on the motion of the former will also apply to the latter.

A closer examination of the entropically penalized solvation shell hypothesis has been reported recently,^64^ which suggested that the loss of independent motion caused a significant decrease in both the heat capacity (*C*_p_) and entropy (Δ*S*) of the bound water relative to free water. Thus, it appears that a thermodynamic explanation can be given for fibroin gelation in NSF at elevated temperatures or following freezing. Nevertheless, while the hydration shell is also likely to play an important role in flow-induced gelation, a complete explanation for this cannot be given at present.

It is also widely recognized that flow can produce nonuniform concentrations in polymer solutions.^65−75^ A number of different explanations for this phenomenon have been suggested, focusing on chain deformation, normal stress, and the amplification of transient local concentration fluctuations that may arise spontaneously under quiescent conditions. While these studies have examined the effect of flow on the polymer chains, however, to the best of our knowledge, none have considered how that might affect associated solvation shells.

The work reported here investigated flow-induced gelation of NSF in the absence of thermal changes. The effects of flow on protein chain deformation, orientation, and phase separation were explored using rheometry in combination with polarized light microscopy (PLM) and infrared (IR) spectroscopy. The results showed that flow caused chain deformation and orientation, producing a significant increase in the first normal stress difference (*N*_1_) and birefringence in the NSF, which was optically isotropic under initial (quiescent) conditions. This was closely followed by microphase separation and gelation, which continued to develop after the flow ceased. Significantly, IR spectroscopy revealed features consistent with loss of the hydration shell, as well as changes in the fibroin conformations from helical or random coil to antiparallel β-sheet structures, in response to flow.

## Experimental Methods

All experiments were performed using NSF specimens freshly taken from the middle-posterior sections of silk glands dissected from commercially bred *B. mori* silkworms in their 5th instar, at the earliest stages of cocoon construction, as described previously.^35,64,76−78^

### Rheology Measurements

The majority of rheology measurements were performed at 25 ± 1 °C, using a DHR2 rheometer (TA Instruments, New Castle, DE, USA), fitted with a CP1/20 cone geometry (20 mm diameter, 1° opening angle, and 27 μm truncation). The specimen temperature was controlled using a Peltier base plate.

As in our previous work,^35,76−78^ sufficient NSF was used to slightly overfill the geometry. Then, after slowly lowering the cone into place, the excess was not removed, as the associated stress can initiate gelation. A water flood was applied around the overfilled specimen and a home-made environmental chamber was used to prevent drying and “skin” formation. While overfilling and applying the water flood may not be ideal, previous work^77^ has shown this to be an effective approach for performing rheology experiments on NSF specimens. In particular, only a small decrease in dynamic moduli was observed at 25 °C over 50 min, which exceeded the timescales required for the experiments performed here.

First, the specimen was conditioned by applying a steady shear rate (γ̇) of 1 s^–1^ over 100 s and the viscosity at this shear rate (η_1_) was obtained as the average over the final 30 s. Then, the oscillatory response was measured over a logarithmic angular frequency (ω) sweep from 125.7 to 0.13 rad s^–1^, at 1% strain amplitude, which was well within the linear elastic limit of the NSF. Finally, experiments were performed as described in the text.

### Polarized Light Microscopy

PLM measurements were performed using a modular microscope attachment on the DHR2, with LED illumination of wavelength 470 nm (Thorlabs, New Jersey, USA). A ×4 objective lens was used, with a capture time of 0.03 s per frame, at 5 frames per second. In this case, the temperature was controlled through the cone, using an upper heated plate attachment. In order to improve their presentation, the sets of raw images were reprocessed consistently, using ImageJ (NIH) to optimize the contrast range and apply the “Fire” false color table.

### Rheo-Infrared Measurements

Combined rheological and IR spectroscopy measurements were performed at ambient temperatures (25–30 °C), using a MCR502 rheometer (Anton Paar), fitted with a parallel plate geometry (PP12, 12 mm diameter) at a 500 μm gap setting. An attenuated total reflectance (ATR) device (Golden Gate, single bounce, 45° incidence angle, with diamond internal reflection element (IRE), SpecAc, UK) used as the bottom plate of the rheometer was incorporated in the IR spectrometer optical path. IR spectra were collected (as 4 scans at 4 cm^–1^ resolution, giving 1.6 s per spectrum) using an IS-50R spectrometer (Nicolet, ThermoFisher Scientific, Waltham, MA, USA), fitted with a liquid N_2_-cooled 1850 mercury cadmium telluride (MCT) detector. In order to collect polarized IR spectra, a motorized filter (WP25H-Z 25mm diameter holographic wire grid on ZnSe, Thorlabs, New Jersey, USA) was incorporated into the optical path.

## Results

### Description of Native Silk Feedstock

In order to better understand the results from the experiments reported here, it is useful to first provide a brief description of NSF from *B. mori* silkworms. The material taken from the middle-posterior silk gland is an aqueous protein solution, containing around 25% w/w of fibroin, with traces of other proteins, sugar (trehalose), and (mainly Ca^2+^ and K^+^) salts, with close to neutral pH (6.5–7.2).^76,78^

Based on published genome analyses,^80−85^*B. mori* fibroin is a dimer of a heavy and light chain (Fib-H and Fib-L), conjoined by a disulfide (Cys–Cys) bond, giving essentially a linear protein, consisting of 5525 amino acids (formula weight around 419 kDa). This protein is largely composed of highly repetitive (GAGAS)_*x*_ sequences interspersed by short sequences of more diverse composition and capped by nonrepetitive sequences at the C- and N-termini. More precisely, due to the position of the disulfide linkage (72 amino acids from the C-terminus of Fib-L and 20 amino acids from the C-terminus of Fib-H), however, the fibroin chain is slightly branched, with arms of 20 and 72 amino acid lengths located at 190 amino acids from one end of a linear 5434 amino acid chain.

Previous studies^64,86^ have shown that fibroin in dilute aqueous solution is essentially a random coil, with its radius of gyration or hydrodynamic radius around 12 nm. Hence, based on the composition NSF and the formula weight of fibroin, simple calculations reveal that the chains are extensively interpenetrating and overlapped, which is consistent with previous estimates of 6–12 entanglements per chain.^76,79^ Moreover, as the Ca ions can bridge between carboxylate-substituted amino acid side chains (Asp and Glu) on the protein, NSF represents a natural example of sticky reptation,^79^ which increases the susceptibility of fibroin toward flow-induced deformation.^37^

### Characterization of NSF and Phase Separation during Shear Rate Ramps

**Figure 1:**
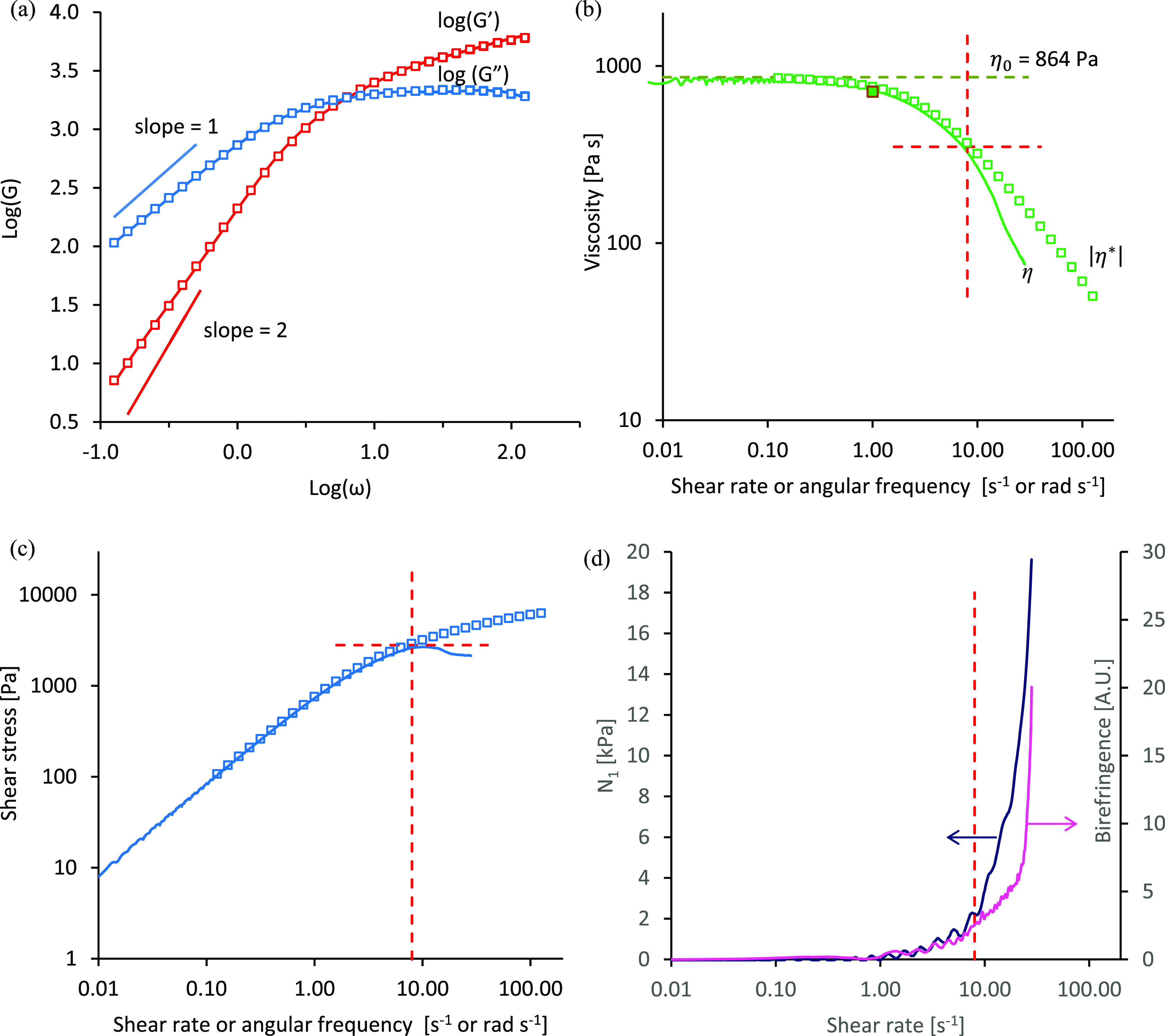
Rheological characterization of an exemplar NSF specimen at 25 °C. (a) log(*G*′) and log(*G*″) vs log(ω) (red and blue squares, respectively) from an oscillation frequency sweep (dynamic moduli in Pa, angular frequency in rads s^–1^); continuous lines represent the best model fit using eq 1a and 1b, with the values shown in Table 1; straight lines represent the expected gradients of 2 and 1 for *G*′ and *G*″ (i.e., indicating ω^2^ and ω dependence) in the terminal region. (b) Open squares are complex viscosity vs angular frequency, from oscillatory data using eq 3; filled square is the initial shear viscosity measurement at γ̇ = 1 s^–1^; continuous line is shear viscosity from the shear rate ramp measurement; dashed green line is η_0_ from oscillatory data using eq 2. (c) Shear stress vs rate: open squares are calculated from the complex viscosity (from oscillatory data) using eq 4; continuous line is shear stress from the shear rate ramp measurement. (d) First normal stress difference (*N*_1_, dark blue) and birefringence (magenta) vs shear rate. Dashed vertical red lines in (b–d) mark the shear rate (8 s^–1^) above which larger deviations between continuous shear and oscillatory measurements and the peak in shear stress were observed; the horizontal lines in (b,c) mark the corresponding viscosity (350 Pa s) and shear stress (2800 Pa).

Rheological characterization of an exemplar NSF sample undergoing flow-induced gelation is shown in Figure 1. Oscillatory measurements of elastic and viscous moduli (*G*′ and *G*″) against angular frequency showed the classic viscoelastic response of a polymer solution (Figure 1a), with *G*′ dominant at high frequency and a crossover to *G*″ becoming dominant at low frequency.^42,44,47^ Considerable sample-to-sample variation has been noted previously,^34,35,76,77^ which has been ascribed to changes in the ionic content of the NSF as the silkworm prepares for and progresses through cocoon construction.^78,79^ Nevertheless, the viscoelastic behavior observed was typical of NSF^35,37,76,77^ and suggests that it represents a conventional polymer solution.

It may be noted that the plots followed the expected ω^2^ and ω dependence of *G*′ and *G*″ at low frequency, indicating that the measurements shown here extended into the “terminal” frequency range, whereby the rheology was dominated by the slowest relaxation mode. Also, consistent with previous observations,^35,76,77^ the present data could be fitted very well using the Maxwell model of viscoelastic behavior^42,44,47^
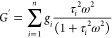
1a
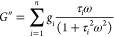
1busing four modes (*n* = 4), which relate to the relaxation behavior of the protein at different length scales. Model fitting allowed the characteristic relaxation times (τ_*i*_) and modulus contributions (*g*_*i*_) to be evaluated; for the exemplar specimen shown in Figure 1, the results from fitting eq 1a are shown in Table 1.

**Table 1:** Fitting Parameters (Relaxation Times and Modulus Contribution) Obtained by Fitting eq 1a and 1b to Oscillatory Data for the Exemplar NSF Sample Shown in Figure 1

	relaxation times [s]	modulus contributions [Pa]
mode 1	3.851	18.12
mode 2	0.352	1590.29
mode 3	0.078	2509.32
mode 4	0.011	3357.35

Thus, the results from fitting the Maxwell model to oscillatory data allowed the zero shear rate viscosity (η_0_ = 864 Pa s for this exemplar) to be calculated using
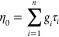
2

The modulus of complex viscosity was also obtained from the dynamic moduli using
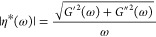
3

A low shear rate Newtonian plateau (up to around 0.8 s^–1^ or 0.8 rad s^–1^ for this exemplar NSF) was observed, above which the viscosities decreased and shear thinning occurred (Figure 1b). Very close agreement was found between η_0_ (calculated from oscillatory data using eq 2) and the instantaneous steady shear viscosity  from a shear rate ramp experiment at low shear rates. Also, good agreement was found between the steady shear and complex viscosities at low rates, corroborating previous observations^34^ that NSF followed the empirical Cox–Merz relationship^87,88^ up to a point (Figure 1b). Beyond this point (around 8 s^–1^ or 8 rad s^–1^, for this example), however, a distinct deviation was observed, with η(γ̇) falling below |η*(ω)|, suggesting that the NSF had undergone a phase change. Based on other results in the present study (vide infra), this was probably due to the separation of water from the protein, causing shear banding or wall slip. The measurements shown here were stopped just as the shear viscosity fell below the complex viscosity; however, previously published results^34^ (and corroborated by other data in the present study) showed that the negative deviation from the Cox–Merz relationship was followed promptly by a steep increase in shear viscosity and first normal stress difference (*N*_1_), suggesting that the NSF had undergone gelation.

The departure from the Cox–Merz relationship can also be observed in terms of the shear stress (Figure 1c), which is related to instantaneous steady shear viscosity through
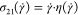
4where the subscripts (1 and 2) refer to the flow and velocity gradient directions. An analogous relationship, with ω replacing γ̇ was used to estimate the corresponding stress from the oscillatory measurements. Here, the onset of deviation from the Cox–Merz relationship coincided with an apparent peak followed by a decrease in shear stress from the steady shear measurements.

Most importantly, it should be noted that a peak in shear stress vs shear rate (i.e., producing a curve that is concave downward) is inconsistent with uniform flow behavior (hence, the term “apparent”). Instead, a peak suggests that the overall shear stress (as measured by the rheometer) could be reduced by the test material separating into two or more layers, which experience higher and lower shear rates (i.e., shear banding^65−75^ or wall slip).

Flow above a shear rate of about 1 s^–1^ (i.e., roughly corresponding to the onset of shear thinning) also produced concurrent increases in birefringence and first normal stress difference
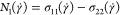
5which is related to extensional viscosity (equal to *N*_1_ divided by the extension rate^89^) and is manifest as the net axial force pushing the rheometer geometry (i.e., the probe attached to the rheometer) away from the plate (Figure 1d). Although measured independently (via the normal force transducer on the rheometer and the camera fitted to the microscope attachment), both effects can be ascribed to chain stretching and segmental orientation. Normal stresses originate from the pressure exerted as chains that have been elongated by flow attempt to retract into more entropically favorable configurations.^42,44,89−91^ Birefringence requires orthogonal differences in the interactions of light with the material (i.e., requiring different polarizabilities or refractive indices), which may arise through several different mechanisms:^41,42,44,47,91−98^ stress birefringence can occur due to changes in polarizabilities when bonds are stretched; orientational birefringence can occur when bonds with different polarizabilities are preferentially orientated in different directions; and structural birefringence can occur due to phase separation into oriented microdomains with different polarizabilities. It was found that *N*_1_ and birefringence increased similarly (albeit, plotted on different scales) between shear rates of about 1–8 s^–1^, suggesting that a constant stress-optical coefficient held within this range, but deviated at higher shear rates (Figure 1d), which may indicate changes in the underlying mechanisms—possibly suggesting microphase separation.

### Orientation Shown by Polarized Light Imaging

**Figure 2:**
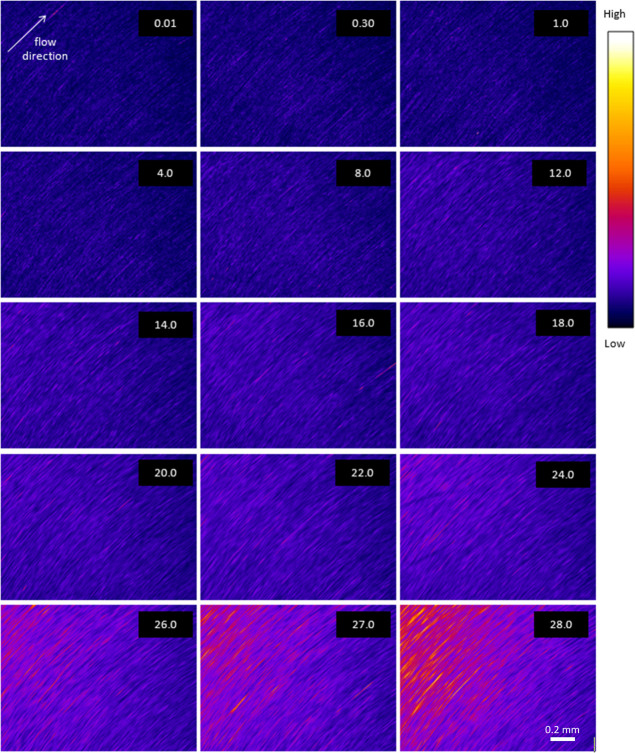
Polarized light microscopy images (corresponding to the NSF specimen in Figure 1), showing increasing birefringence during a shear rate ramp (the image labels indicate shear rates in s^–1^ when the image was recorded.) A ×4 objective lens was used throughout, giving a field of view of 1.6 × 1.2 mm for each image; a scale bar is shown in the final image. The microscope was offset 3 mm “West” and 3 mm “North”, from the center of the cone geometry, and the polarizers were set at roughly ±45° to the flow, so that the images were expected to be dark in the absence of orientation, becoming lighter as flow-induced birefringence increased. False color (“Fire” color table in ImageJ) has been applied, with dark blue indicating lowest and white indicating the highest intensity levels. Numerical data for the transmitted light intensities corresponding to these images are plotted in Figure 1d.

The gradual increase in birefringence with the shear rate is also demonstrated by the changing colors in the PLM images (Figure 2). At shear rates below 1 s^–1^, the images were essentially featureless, with minimal birefringence (i.e., purple, with the “Fire” false color table applied, using ImageJ). The fine “background” texture of lighter spots and short lines running roughly diagonally in the images are due to residual “tooling marks” on the rheometer geometry. These marks remained visible in images at faster shear rates, but above 8 s^–1^, became considerably blurred by movement during the capture time (0.03 s) of each frame. Based on the 1° opening angle of the cone geometry used and the offset of the camera from the center (3 mm “West”, 3 mm “North”), it can be estimated that the part of the geometry in the field of view moved by around 18 μm (equivalent to around 7 pixels) during each frame at a shear rate of 8 s^–1^; this increased to around 63 μm movement (equivalent to around 25 pixels) at the final shear rate of 28 s^–1^.

The increase in birefringence became more obvious (diagonal features became lighter, trending toward orange) above a shear rate of 8 s^–1^, which coincided with the value above which deviation from the Cox–Merz relationship was observed (Figure 1b,c). Birefringence also became more prominent in the upper left quadrant, probably because the optical path length through the NSF specimen was longest in that part of the image. Nevertheless, while the tooling marks became more prominent as birefringence increased, no other structures were observable in the images.

**Figure 3:**
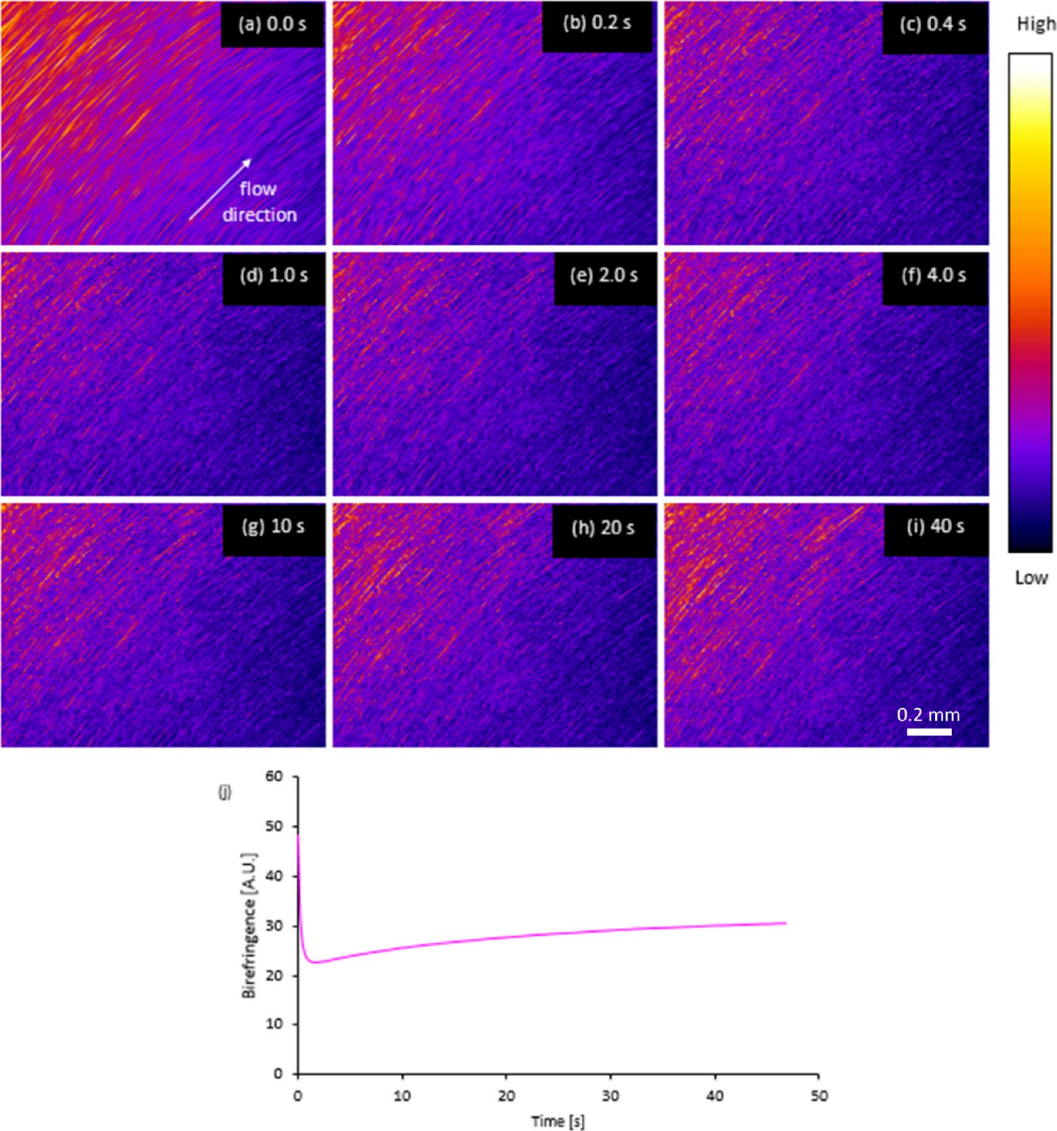
Changes in birefringence in a (nominally quiescent) specimen following a shear rate ramp (corresponding to the NSF specimen in Figure 1) (a–i): sequence of polarized light images at the times shown; the magnification was the same as in Figure 2 and a scale bar is shown in the final frame. (j) Changing birefringence (transmitted light intensity, in arbitrary units) vs time after shear flow cessation.

Once the target shear rate (28 s^–1^) had been achieved, the rheometer stopped while imaging continued for a further 47 s (Figure 3). The first image (at 0 s) coincided with the end of the shear rate ramp and was still considerably blurred due to the controlled movement of the rheometer. The second image (at 0.2 s) was also slightly blurred due to uncontrolled movement driven by the residual stress in the sheared NSF specimen, as the geometry was allowed to move freely in this part of the experiment. This (reversed) movement continued more slowly during the rest of the images, consistent with previous observations^24,35,76^ that stress relaxation can continue over several tens to hundreds of seconds.

An interesting change in birefringence was observed during the relaxation of the specimen after the shear flow was stopped, as demonstrated by the images (Figure 3a–i) and the intensity plot (Figure 3j). Initially (during the first 2 s), the birefringence decreased sharply as the shear flow driven by the rheometer ceased, consistent with the (nominally quiescent) NSF specimen undergoing relaxation.

Subsequently, however, the birefringence increased slowly, demonstrating that further morphological changes were occurring. As the specimen was effectively quiescent during this part of the experiment, however, it seemed unlikely that its orientation increased. Instead, in view of the various mechanisms by which birefringence can increase,^41,42,44,47,91−98^ it seemed more likely that the increase observed here may have been due to changes in solvation (affecting bond polarizabilities) or sub-microscopic morphology (i.e., structural birefringence).

Although these data did not confirm that the NSF specimen had gelled, they were consistent with previous observations^35^ that, once initiated, flow-induced changes can continue after the flow has ceased. Again, the tooling marks on the geometry were visible, but no other structures were evident in the images. This suggests that the characteristic length scale of whatever was responsible for the birefringence was below the resolution (around 7.5 μm, corresponding to 3 pixels) of the relatively low (×4) magnification objective lens used on the camera.

### Comparing Gelation Triggers in Similar NSF Specimens

**Figure 4:**
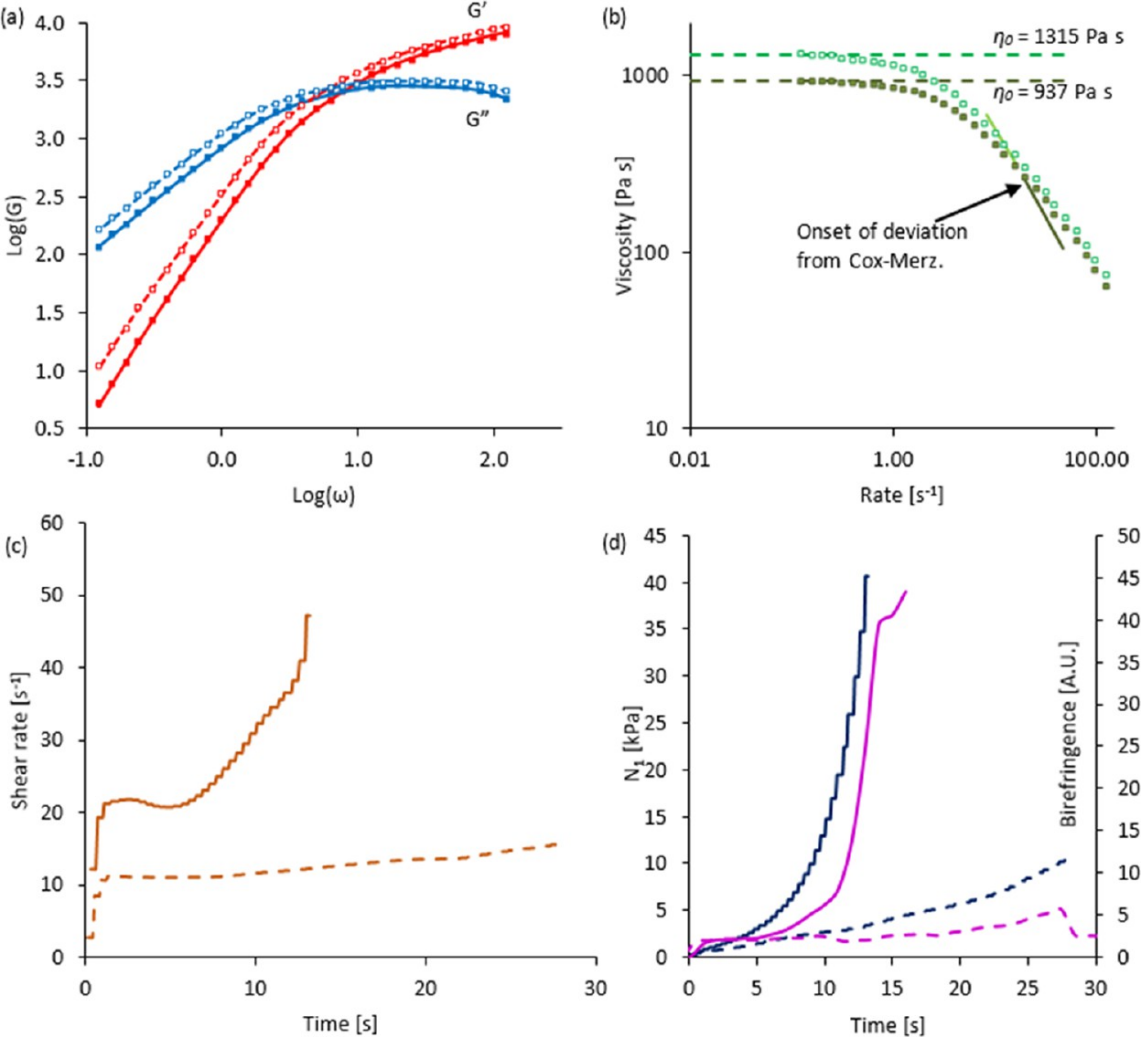
Comparison of rheological data between a specimen that gelled (filled symbols or solid lines) and a similar one that did not (open symbols or dashed lines) during nominally identical experiments using a 5 kN shear pulse. (a) Dynamic moduli from oscillatory sweeps. (b) Complex and steady shear viscosities vs shear rate or angular frequency, arrow indicates onset of deviation between |η*| and . (c) Shear rate vs time (numerical integration of the rheometer data gave total strains of 347 and 334 for the ungelled and gelled specimens, respectively). (d) First normal stress difference (*N*_1_, dark blue) and birefringence (magenta) vs time.

Sequential rheological measurements are compared between two approximately similar specimens from different silkworms, one of which underwent gelation (shown by filled symbols or solid lines in Figure 4), while the other did not gel (open points and broken lines) during nominally identical experiments. Natural variation between silkworms and changes prior to spinning^34,35,76,77^ made it difficult to select exactly identical NSF specimens. While the fibroin chain lengths were under genetic control and the concentrations were generally similar (around 25% w/w), it has been found that the viscosity is strongly affected by the ratio of K^+^ to Ca^2+^ ions. Nevertheless, initial characterization of the specimens selected, using steady shear and oscillatory measurements gave roughly similar values of viscosity and plots of dynamic moduli (Table 2, Figure 4a,b), suggesting similar ion concentrations.

**Table 2:** Rheological Data for the Exemplar NSF Samples Shown in Figures 4 and 5^a^

	sample gelled	sample did not gel
η_1_ [Pa s] from steady shear	738	907
η_0_ [Pa s] from oscillatory data	937	1315

	relaxation times [s]	modulus contribution [Pa]	relaxation times [s]	modulus contribution [Pa]
mode 1	2.99	30.16	2.16	43.49
mode 2	0.356	2053.9	0.287	2353.9
mode 3	0.079	3443.6	0.068	3648.6
mode 4	0.013	3848.9	0.012	4611.1

a Relaxation times and modulus contributions were obtained by fitting eq 1a and 1b to oscillatory data; the zero shear rate viscosity was obtained from those values using eq 2.

Curve fitting using eq 1a and 1b produced slightly higher values of modulus contributions and relaxation times for the specimen that did not gel, giving a higher value of zero shear rate viscosity (1315 Pa s), compared with the other (937 Pa s) that did gel.

Subsequently, a stress-controlled (σ_21_ = 5 kPa) shear pulse was applied to each specimen, up to a set time limit (28 s) or until a first normal stress difference limit of 40 kPa was exceeded. The higher viscosity specimen underwent steady flow (at γ̇ = 10–16 s^–1^, corresponding to a decrease in shear viscosity from 500 to 315 Pa s, Figure 4b,c), with small increases in *N*_*1*_ and birefringence during 28 s of shear flow (Figure 4d). By contrast, the lower viscosity specimen underwent a relatively fast increase in the shear rate (up to γ̇ = 47 s^–1^, corresponding to a decrease in shear viscosity from 260 to 106 Pa s), with similarly rapid increases in birefringence and *N*_1_ to the set limit, triggering the end of this stage of the experiment (at 13 s). Interestingly, comparing the complex viscosities measured before the shear pulse with the shear viscosities measured during the shear pulse (solid lines in Figure 4b) produced a close match for the former (ungelled) specimen (i.e., following the Cox–Merz relationship until the end of the flow period), while a clear deviation was found for the latter (gelled) specimen, with  falling below |η*(ω)|, suggesting microphase separation, similar to the behavior shown in Figure 1.

**Figure 5:**
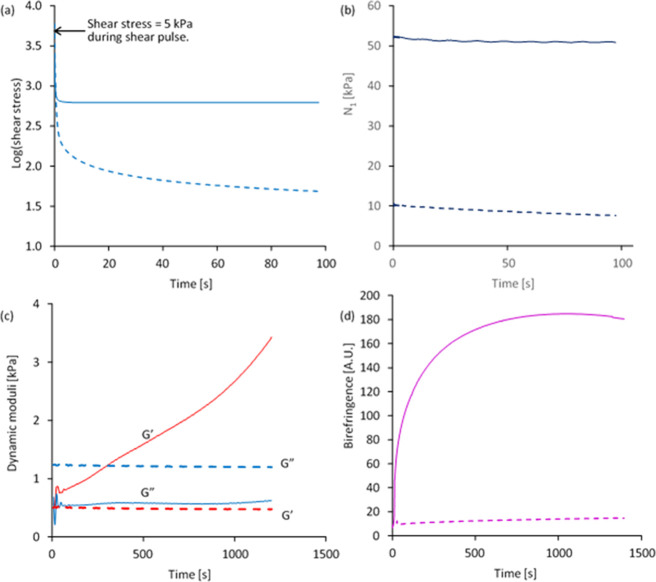
Changes following the cessation of shear flow for a NSF specimen that gelled (solid lines) and one that did not (dashed lines) during nominally identical experiments (continuation of the data in Figure 4): (a,b) relaxation of shear stress and first normal stress difference over first 100 s following cessation of shear flow (the arrow indicates the (5 kPa, set) shear stress during the shear pulse, prior to relaxation.); (c,d) changes in dynamic moduli (*G*′ in red, *G*″ in blue, measured at 1% strain amplitude and an angular frequency of 1 rad s^–1^) and birefringence (purple) vs time. The data shown in (c) follow the initial 100 s relaxation period covered in (a,b); data in (d) are from the start of the experiment and includes the periods of shear flow and relaxation.

Immediately following the end of the shear pulse, stress relaxation was measured—in this case, with the geometry position held. The shear stress of the higher viscosity specimen (that had flowed steadily) decreased considerably to a residual shear stress of 49 Pa after 100 s (equivalent to log(σ_21_) = 1.6873, dashed line in Figure 5a). At the same time, about half of the birefringence that had developed in this material during the shear pulse was rapidly lost (magenta dashed line in Figure 4d). By contrast, the other specimen showed an initially rapid relaxation, but then maintained a residual shear stress of 618 Pa at 100 s (equivalent to log(σ_21_) = 2.7910, solid line in Figure 5a). In this case, the birefringence continued to increase, albeit at a lower rate, after flow ceased (magenta solid line in Figure 4d).

Rather different behavior was observed for the first normal stress differences (Figure 5b). The higher viscosity sample attained a relatively small value of *N*_*1*_ (10 kPa), while a much higher value (52 kPa) was attained by the lower viscosity sample. In contrast to the shear stress, however, values of *N*_1_ decreased relatively very little for either sample over 100 s. This suggested that flow induced extensional strain in the protein chains, which relaxed relatively slowly after flow stopped. This seems rather surprising, as it may be expected that shear and normal stresses would be affected by the same molecular motions. Consequently, this observation cannot be explained at present.

As noted previously,^24,35^ a high level of residual shear stress suggested gel formation, which was confirmed by measuring the changes in dynamic moduli over the subsequent 20 min (Figure 5c). A previous work^35^ showed that gelation was accompanied by an increase in *G*′, particularly at low frequency, until it was above *G*″ across the entire frequency range.

The more viscous specimen (that had flowed steadily) remained in a more liquid state with essentially constant *G*″ > *G*′ at 1 rad s^–1^. The other was dominated by elastic behavior with progressively increasing *G*′ > *G*″, showing that the gel structure continued to develop under effectively quiescent conditions (i.e., 1% oscillatory strain amplitude at an angular frequency of 1 rad s^–1^).

**Figure 6:**
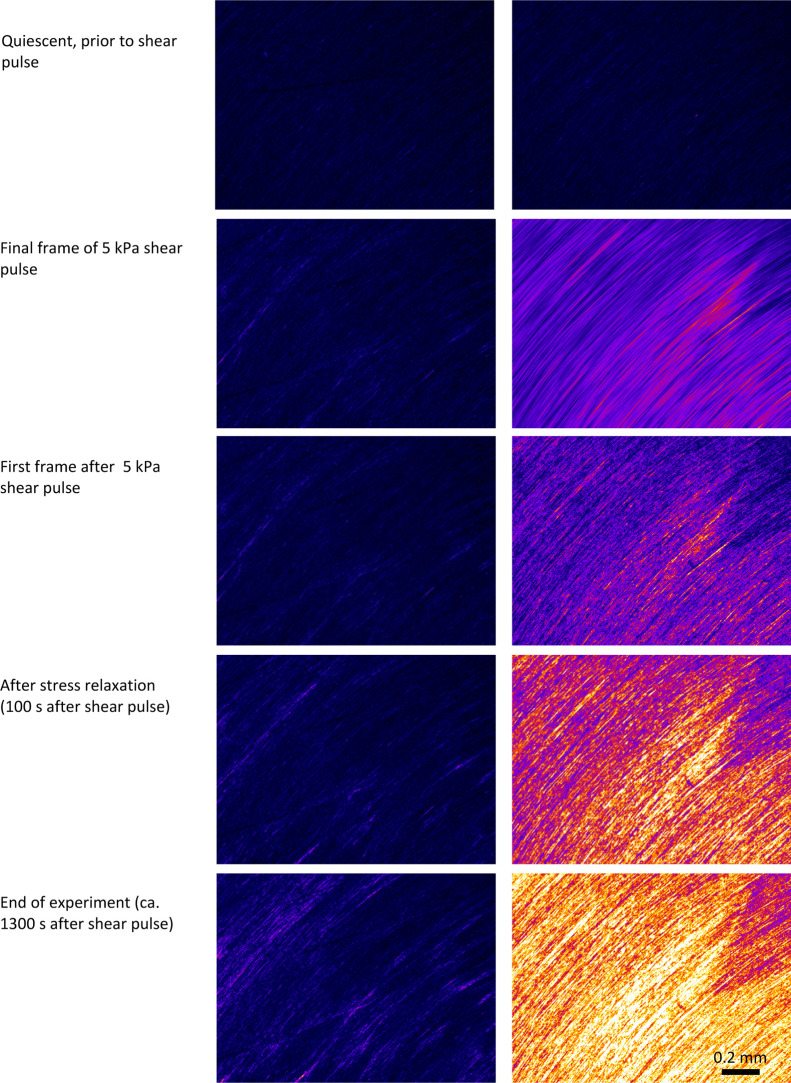
Sequence of polarized light images (corresponding to the NSF specimens in Figures 4 and 5, at different stages of the 5 kPa shear pulse experiments). Images on the left represent the higher viscosity specimen that did not gel during the experiment (extensive stress relaxation occurred following the shear pulse and *G*′ < *G*″ at ω = 1 rad s^–1^ during the subsequent 20 min). Corresponding images on the right are from a similar specimen that did gel during the experiment (*G*′ > *G*″). As in Figure 2, images were collected from the “North–West” quadrant, such that flow induced orientation and birefringence. The specimens remained at essentially the same positions in all these images (i.e., rheometer held its position with no driven flow), from the first frame after the shear pulse to the end of the experiment. The magnification was the same as in Figure 2 and a scale bar is shown in the final frame.

Progressive changes in gel structure during the subsequent quiescent period were also revealed through birefringence measurements (Figures 5d and 6). The ungelled specimen showed a slight increase in overall birefringence, while examination of the PLM images (Figure 6) revealed elongated strands of lighter colors gradually appearing. These strands may have been similar to the fibrils reported previously in sheared NSF specimens.^36^ By contrast, the gelled specimen became considerably more birefringent, with several discrete elongated structures apparent in these images.

It should be emphasized that little or no overall flow was expected beyond the end of the shear pulse (only the oscillation required to measure the dynamic moduli). In this case, the rheometer was programed to hold its position from the end of the flow stage, which can be confirmed by comparing the positions of various features in the images. Again, it seemed unlikely that the subsequent increase in birefringence could be due to further flow-induced deformation of the gel; instead, changes in solvation (affecting bond polarizabilities) or sub-microscopic morphology (i.e., structural birefringence) may have been involved.

### Specific Work Rate

Previous studies^35^ suggested that exceeding a shear stress threshold (between 5 and 12 kPa) led to gelation. Thus, the stress pulses applied here (at σ_21_ = 5 kPa) were expected to be close to the minimum threshold for initiating gelation. It has also been suggested^34^ that the energy density input (or specific work, *w*) during flow provided a more appropriate criterion, by combining the shear rate, shear stress, and time. In the present case, however, numerical integration over the shear pulse using
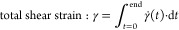
6a
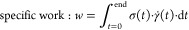
6b

gave similar values for both the total shear strain (334 and 347) and specific work (1.5 and 1.6 J g^–1^) for the two experiments—with the slightly lower values for the sample that went on to gel. Thus, in spite of the apparent similarities between the two NSF specimens, it may be informative to explore their differences. Perhaps the most important difference was the way their initial viscosities and the subsequent shear thinning at constant shear stress led to very different shear rates (Figure 4c). Moreover, since the flow occurred at constant shear stress, the specific work rates were considerably different: up to 0.21 J g^–1^ s^–1^ for the sample that gelled, while the other remained below 0.075 J g^–1^ s^–1^. To put these findings in context, recent extensional rheology studies^99^ found that fibers could be produced by drawing at specific work rates above 0.1 J g^–1^ s^–1^, with higher rates producing greater chain orientation and stronger fibers.

The other obvious differences were in the development of normal stress and birefringence (Figure 4d). As both of these parameters may be related to chain stretching,^41,42,44,47,89−98^ however, it is likely that these differences were consequences of the different shear or work rates.

### Observations from Rheo-IR Spectroscopy

**Figure 7:**
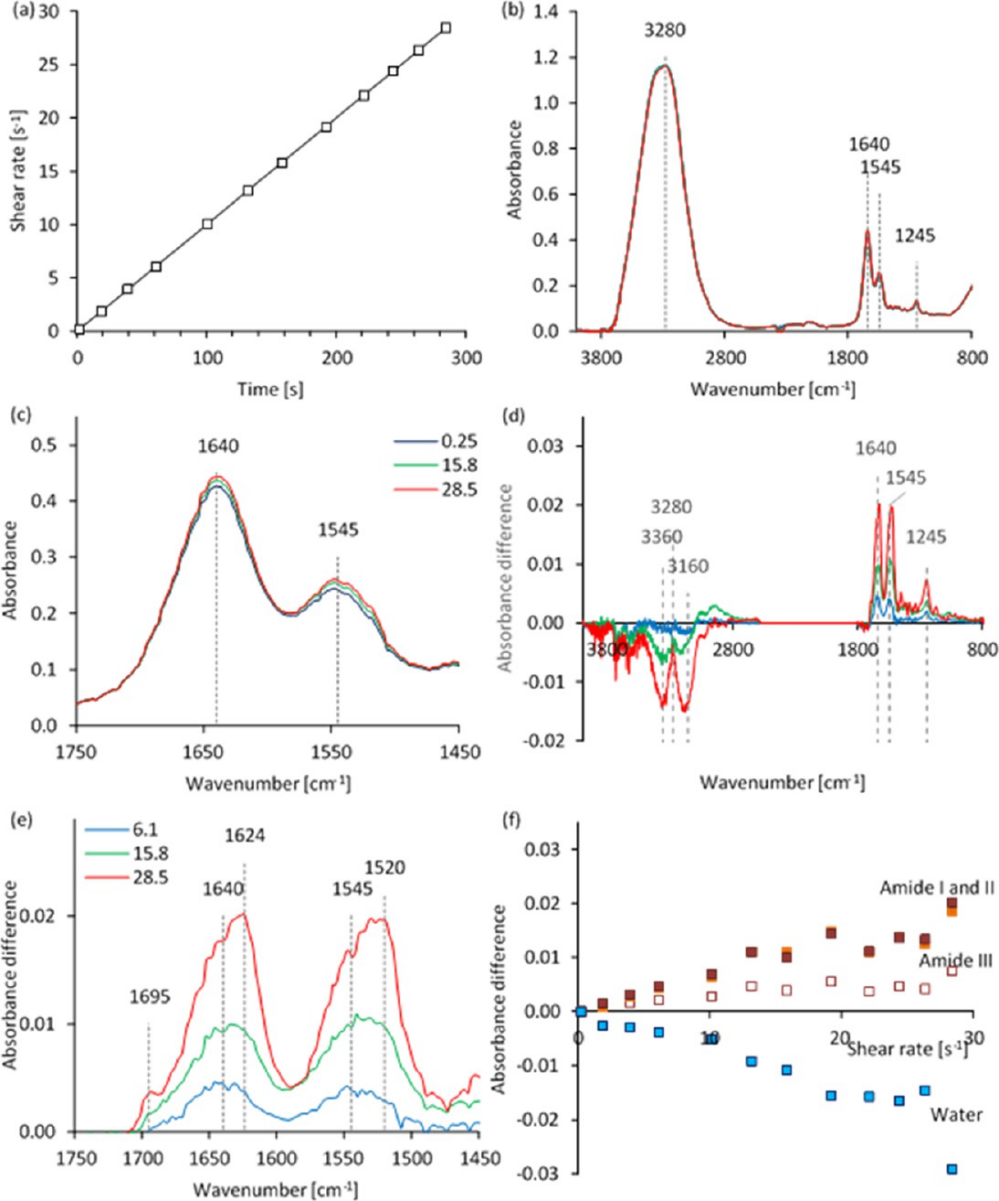
Unpolarized rheo-IR data from an exemplar NSF specimen, during a linear shear rate ramp at 25 °C. (a) (Nominal) shear rate profile; the open squares mark when spectra were collected. (b,c) Spectra collected at the shear rates (in s^–1^) shown in (c). (d,e) Difference spectra over full-range and for the amide I and II regions (after subtraction of the initial spectrum at 0.25 s^–1^), at the shear rates shown in (e). (f) Differences in intensity from the initial spectrum, for amide I (brown), amide II (orange), amide III (open symbols) and water bands (average calculated from the troughs at 3160 and 3360 cm^–1^, blue).

While flow-induced phase separation in NSF specimens could be inferred from the rheological and PLM data, it was found to be more obvious in IR spectra. In a typical experiment (Figure 7), spectra were collected during a linear shear rate ramp, with the NSF specimens on an ATR stage. Although shear rates are quoted (Figure 7a), the use of a parallel plate geometry with the rheo-IR apparatus meant that the values were only nominal (corresponding to the maximum value at the edge of the geometry) and varied across the region detected by the ATR.

Cursory examinations of spectra for a NSF specimen (Figure 7b,c) revealed four major absorbance bands that can be ascribed to the amide I, II, and III bands of the peptide groups (around 1640, 1545, and 1245 cm^–1^),^100−102^ O–H stretching (around 3280 cm^–1^), and water H–O–H bending (around 1645 cm^–1^, overlapping the amide I band).^103−106^ Closer scrutiny also revealed other less intense bands due to the protein, in the “fingerprint” region between 1540 and 900 cm^–1^. Flow produced only very small overall changes (less than around 5% of peak heights), which were difficult to observe in the full-range spectra (Figure 7b) but became clearer by examination of selected regions (e.g., the amide I and II region, Figure 7c) or difference spectra (i.e., obtained by subtraction of the initial spectrum at 0.25 s^–1^, Figure 7d,e).

It was postulated that the torque transmitted to the ATR stage during shear flow may have caused it to move slightly, and the resulting change in alignment could have affected the intensities of the spectra. During the experiment, however, it was found that the protein bands in the difference spectra increased in intensity, while the water O–H stretching band became negative. This changing balance between the O–H stretching (due to water) and the N–H (due to protein) bands produced the two troughs (around 3360 and 3160 cm^–1^) on either side of the amide A peak (the stronger band of a Fermi doublet, around 3280 cm^–1^).^100−102^ Since a change in ATR stage alignment would be expected to affect the absorbances due to water and protein similarly, it did not appear to be a viable explanation.

The changes in absorbance due to water and protein increased in magnitude as the flow rate increased (Figure 7f). Since the ATR method^52,107−110^ depends on electromagnetic interactions within a few micrometers from the surface of the IRE, the changes in peak height suggested that fibroin was attracted toward the apparatus, while water moved away. Similar observations from repeated experiments (15 out of 19) suggested that this reflected the most frequent behavior, although the opposite was found in some (2/19) cases: water moved toward the IRE and fibroin moved away. Moreover, in a few cases (2/19), the changes appeared to start in one direction before reversing. It was not possible to make any quantitative estimates of the associated composition changes, however, as the spectra represented the total material within the irradiated sample volume, while the numbers, sizes, shapes, compositions, and positions of microphase domains relative to the IRE were not known.

In addition to the changes in peak height associated with microphase separation, changes in the apparent positions of the amide I and II difference peaks were observable (Figure 7e). In principle, peptide bands can be deconvoluted to provide structural information.^100−102,111−119^ In addition to chain conformation, however, it is expected that the peak positions in NSF are also affected by the presence of water, due to its dielectric constant and the strength of hydrogen bonding.^100−102^ Moreover, the peak centered around 1640 cm^–1^ in the direct spectrum of NSF (Figure 7b,c) is expected to include at least two components due to the amide I and water bending bands.^100−106^ It may be noted that additional absorbance due to water accounts for the intensity of the 1640 cm^–1^ peak being roughly twice that of the 1545 cm^–1^ peak; otherwise the amide I and II peaks of *B. mori* fibroin should be of similar height.^52,76,120^

Nevertheless, the difference spectra indicated an increasing protein concentration offset by decreasing water. Hence, the increase in peak height around 1640 cm^–1^ in the difference spectra (Figure 7d,e) may be ascribed to amide I. This band originates predominantly (about 80%) from the peptide C=O stretching vibration and has been commonly used to interpret the protein structure. As the present work investigated the transition of silk protein from aqueous solutions to hydrated gels, however, we cannot be certain to what extent the peak positions were governed by overlap with water (for amide I), the presence of hydration, or morphological factors. Consequently, deconvolution was not attempted.

Nevertheless, the difference peak at 1640 cm^–1^ was consistent with a hydrated random coil or helical configuration. As the experiment progressed, this peak increased in intensity, due to the increasing protein concentration relative to water, and appeared to move to 1624 cm^–1^. In reality, this was probably due to the growth of a peak around 1624 cm^–1^, while the height of the original peak at 1640 cm^–1^ decreased, rather than a bathochromic shift in peak positions. Concurrently, a new peak appeared at 1695 cm^–1^, while the amide II peak (the asymmetric combination of C–N stretching and N–H bending vibrations) appeared to move from 1545 to 1520 cm^–1^ (Figure 6e). These changes are consistent with the loss of random coil or helical structures in NSF and the fibroin developing an antiparallel β-sheet structure in response to flow.^100−102,111−119^

**Figure 8:**
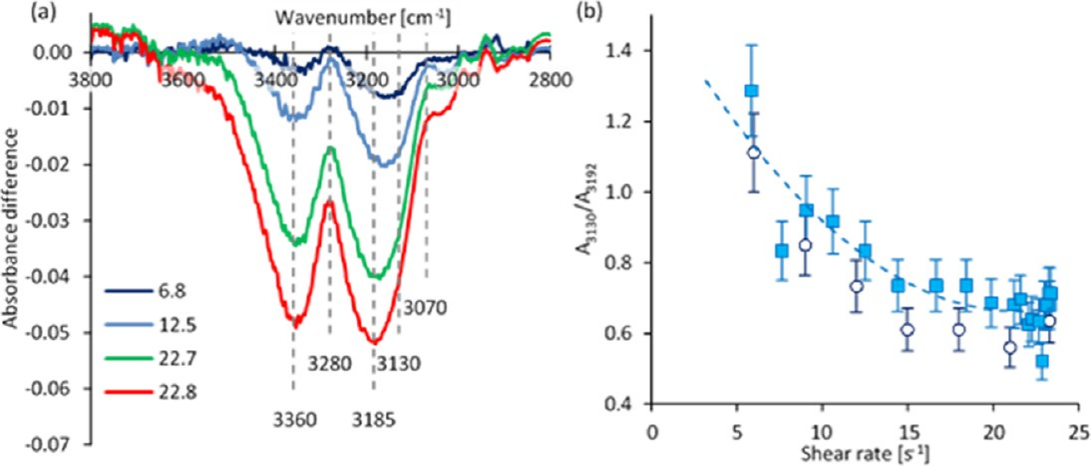
Changes in the O–H and N–H stretching regions during a linear shear rate ramp for NSF specimens at 25 °C. (a) Absorbance differences of unpolarized difference spectra (i.e., after subtraction of the first spectrum from the subsequent spectra) for an exemplar NSF specimen at the nominal shear rates shown (in s^–1^). (b) Intensity ratios of model peaks at 3130 and 3192 cm^–1^; different colors and symbols represent data from duplicate NSF specimens and the dashed line serves only as a guide for the eye.

As the microphase separation increased during the experiment, the weaker amide B band (the second part of the Fermi doublet^100−102^) also became evident as a small positive peak, around 3070 cm^–1^. In addition to the obvious changes in the intensities of the bands above 3000 cm^–1^, due to the relative concentrations of water and protein, more subtle changes in peak position were also found (Figure 8a). Specifically, the negative peak around 3170 cm^–1^ (actually, due to the broad negative water O–H stretching band being partially offset by the positive amide A band) appeared to move to a slightly higher wavenumber (around 3185 cm^–1^). It was found that this part of the spectrum could be modeled by the sum of two Gaussian terms
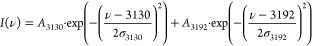
7where ν is the wavenumber, A_3130_ and A_3192_ are the intensities and σ_3130_ and σ_3192_ are the widths (set at 25 and 30 cm^–1^, respectively) for bands centered at 3130 and 3192 cm^–1^. The position of the 3192 cm^–1^ band may not be meaningful, as it resulted from the difference between the broad O–H stretching envelope and the amide A band. The 3130 cm^–1^ band was close to the low wavenumber limit of the O–H stretching envelope, however, and was likely to be less affected by the weaker amide B band; hence, it probably originated from a real component with a frequency around 3130 cm^–1^. It was assumed that the peak positions and widths remained constant but that the intensities could vary. Thus, modeling suggested that the 3130 cm^–1^ band initially dominated in the difference spectrum, but gradually decreased relative to the higher wavenumber band as the experiment progressed (Figure 8b), thereby accounting for the apparent change in peak position.

Attempts were also made to model the entire spectrum between 3000 and 3600 cm^–1^, but this achieved only limited success due to the resulting complexity. It may be noted that, in addition to intensity, each component in the model requires values for wavenumber position and breadth, which were difficult to evaluate accurately due to the overlap between peaks. The modeling was further complicated by slight changes in the baseline above 3600 cm^–1^ or below 2900 cm^–1^ (Figure 7d or Figure 8a). Nevertheless, the results from this more complicated modeling also suggested that the relative amount of the lowest frequency O–H stretching component decreased as the experiment proceeded.

As is well known,^103−106^ stronger H-bonding moves the O–H stretching band to lower frequencies; hence the band around 3130 cm^–1^ can be ascribed to water molecules strongly interacting with adjacent molecules. A previous work^64^ has also suggested that water molecules bond more strongly to peptide groups in NSF than to other (bulk) water molecules (with Δ*H* around −525 J mol^–1^ of water at room temperature). Thus, the band at 3130 cm^–1^ is consistent with the O–H stretching vibration of water molecules in the hydration shell around fibroin molecules. Consequently, as the difference spectra revealed changes from the starting composition, they suggested that water in the hydration shell was lost first, before its signal was masked by a more general phase separation. Moreover, while the loss of hydration from fibroin during silk fiber spinning may be inferred from other work, our rheo-IR data appears to provide a direct observation of the hydration shell being lost in response to flow, during the earliest stages of gelation.

### Observations Using Polarized Rheo-IR Spectroscopy

These observations expand upon those reported previously by Boulet-Audet et al.^111,112^ Those studies reported changes in the polarized IR spectra between 1000 and 1800 cm^–1^, concentrating on the amide I and II bands of the protein. Based on the orientation of the dipole moments of these bands relative to the protein chain, changes in their relative heights were interpreted in terms of flow-induced alignment of the fibroin leading to β-sheet formation. Polarized IR was used exclusively and any changes in the spectra outside of the 1000–1800 cm^–1^ range were not reported.

By contrast, the data shown here (Figures 7 and 8) used unpolarized IR and the entire mid-range (800–4000 cm^–1^) was studied. By not using a polarizing filter, the IR illumination was stronger, allowing faster acquisitions or better signal-to-noise in the spectra. The spectral changes observed suggested flow-induced microphase separation of fibroin from water; however, this methodology precluded obtaining any information on chain orientation. The relative peak intensities observed may also have been slightly affected for oriented specimens, if the illumination was partially polarized due to reflections along its optical path.^121^

Subsequently, although incorporating a polarizing filter reduced the IR intensity, it became possible to investigate molecular orientation within the specimens.^52,111,112,121−123^ In the present experiments, the polarization filter was motorized and its orientation was changed automatically (at roughly 7.5 s intervals) to provide s- or p-polarization (i.e., with the electric vector parallel or perpendicular to the IRE surface). For the configuration of the rheometer and ATR device used, s-polarization corresponded to the flow direction, while p-polarization coincided with the transverse direction.

**Figure 9:**
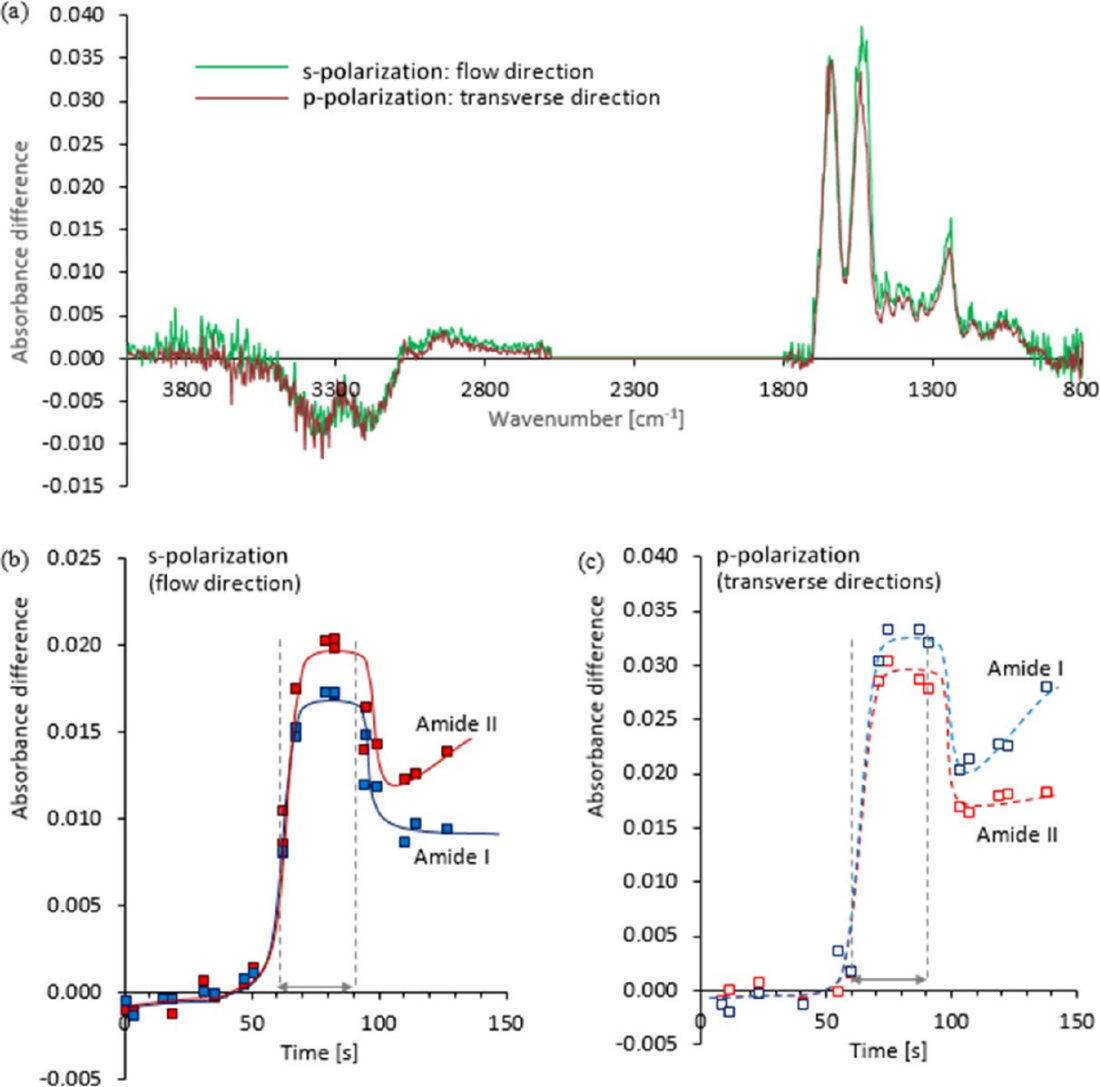
Polarized rheo-IR data from an exemplar NSF specimen at 25° C, subjected to γ̇ = 0 s^–1^, for 60 s, followed by a shear pulse using a (nominal) shear rate of 20 s^–1^ for 30 s, then a second quiescent period. Gray arrows between the vertical dashed lines in (b,c) indicate when the shear flow occurred. (a) Polarized difference spectra, averaged over three scans collected during the shear pulse (between 70 and 90 s), after subtraction of the corresponding initial spectra in s- and p-polarizations, to sample the flow or transverse directions (the s-polarization spectrum has been scaled ×2 to match the amide I intensity in p-polarization). (b,c) Changes in the intensities of amide I and amide II bands measured with s- and p-polarization (differences from the start of the experiment are shown and these intensities were not scaled for the different interactions in s- and p-polarizations).

In order to check whether loading had initiated any changes, spectra were first collected while the NSF specimen remained quiescent for 60 s; then further spectra were collected while a shear rate of (nominally) 20 s^–1^ was applied over 30 s (expected to initiate gelation) and after the shear flow had stopped (Figure 9). No significant changes were observed during the initial 60 s, indicating that flow stresses during loading had not affected the NSF specimen. Subsequently, the start of flow (indicated by gray arrows and vertical dashed lines in Figure 9b,c) was marked by sudden increases in the intensities of the protein bands, with corresponding decreases in the water O–H stretching band. Difference spectra (i.e., spectra obtained by subtraction of the initial s- or p-polarized spectrum) showed positive increases in the amide bands and negative changes in the water band (Figure 9a), suggesting that microphase separation had occurred, with the protein moving toward the IRE and water moving away (similar to the data in Figures 7 and 8). Concurrently, systematic differences were also observed in the relative intensities of the amide bands: the amide II was stronger than the amide I in the s-polarization spectra (i.e., parallel to the flow direction, Figure 9b), while the opposite was found with p-polarization (i.e., parallel to the transverse direction, Figure 9c). Although the amide III band was considered to be too weak to allow reliable analysis, it also became relatively stronger in the s-compared with p-polarization (Figure 9a).

Transition dipole coupling between peptide groups affects both the band positions and dichroism. The strongest component of the amide I band is oriented roughly perpendicular to the peptide chain backbone, while the strongest components of the amide II and III bands are roughly parallel to the chain.^113,119^ Hence, these differences in relative intensities provided clear indications that at least some of the fibroin had been oriented with its chain axis in the flow direction, in agreement with the previous reports by Boulet-Audet et al.^111,112^

Further sudden changes occurred within a few seconds after the end of the flow period, with the amide peaks decreasing and the O–H stretching band increasing slightly. This produced sudden decreases in the amide I and II peaks in the difference spectra (Figure 9b,c), with corresponding increases in the water O–H stretching band. Since the overall band intensities in the direct spectra did not change significantly, partial sample ejection can be discounted. Instead, it appears that some remixing of fibroin and water may have occurred when the flow stopped. Finally, the relative strength of the amide II band increased in the s-polarization (flow direction) spectra (Figure 9b), while the amide I band increased in the p-polarization (transverse direction) spectra (Figure 9c), suggesting that the fibroin became more oriented during further evolution of the gel after flow had stopped. This concurred with the changes observed in birefringence of quiescent material after a shear pulse (Figures 3, 5 and 6).

## Discussion

The combination of rheological data, PLM, and IR spectroscopy presented here provided clear insights into the responses of NSF during flow leading to physical gelation (i.e., without chemical changes). Evidence of flow-induced orientation emerged, and while several mechanisms could (and probably did) account for the observed birefringence, polarized IR indicated that orientation occurred down to the level of peptide groups along the protein chain. At the same time, once a shear rate (or specific work rate) threshold was exceeded, microphase separation occurred, leading to changes in the relative amounts of water and protein observed by ATR-IR spectroscopy. Hence, the deviations from the Cox–Merz relationship and the peak observed in the shear stress at higher shear rates (Figure 1) could have been caused by shear banding into fibroin-rich and water-rich domains or “wall slip” due to flow-induced water migration to the outside of the NSF specimen. This may also have led to changes in the type of birefringence shown by the NSF samples, from stress or orientational birefringence at low shear rates to include structural birefringence at higher shear rates.

The separation of water from protein may play a very important part during silk spinning. As discussed by Sparkes and Holland,^124^ silkworms (and other silk-producing animals) have limited capabilities to extrude the fiber by internal pressure alone; consequently, pultrusion is likely to be involved to some extent. The formation of a water layer between the duct walls and the nascent protein fiber would allow some wall slip and plug flow, thereby further reducing the stress required for the fiber to emerge.

The observations presented here are consistent with much of the previous work and supports the generally held opinion that natural silk fibers are formed by flow-induced gelation. Results presented here and elsewhere^22−25,34−37,99,111,112^ show that gelation can occur simply due to flow, without the need for temperature change or the addition of other agents, although changes in pH and ion content may play important roles in natural silk spinning.^26−32,78^ Moreover, observations of increasing birefringence, normal stress, and differences in polarized IR spectra point to the fibroin chains becoming elongated and oriented by flow prior to gelation. This may also account for the observation that the specific work rate (rather than total work per se) can initiate gelation as diffusion at lower work rates (i.e., reptation at lower viscosities or flow rates) can allow chain relaxation, thereby avoiding any net accumulation of elongation and orientation.

The importance of chain elongation was highlighted by Greving et al.;^125^ using atomic force microscopy, they observed that *B. mori* fibroin chains became elongated and self-assembled into fibrils following shear. Also, a recent work by Asakura and co-workers^126,127^ found that applying modest draw ratios (3–6 times) to native silk specimens (actually, partially coagulated or dried filaments of fibroin solution) caused changes in conformation, with loss of the random or helical structures originally present and increased β-sheet content.^128−132^

Loss of the protein hydration shell during silk fiber spinning may be inferred from much previous work; indeed, separation of water and its recovery by the animal appears to be a common feature in natural silk spinning.^27,78,133−135^ Nevertheless, the rheo-IR data presented here provided a direct indication that the hydration shell can be lost in response to flow alone, during early stages of flow-induced gelation.

Together with loss of the hydration shell, changes in IR spectra indicated the formation of new peptide-to-peptide H-bonds between fibroin chains in β-sheet structures. The strength and regularity of these prevent the fibroin from redissolving in water—although dissolution using more powerful solvents such as aqueous LiBr solution is still possible.^136^ Thus, the fibroin undergoes a physical gelation, without chemical changes.

**Figure 10:**
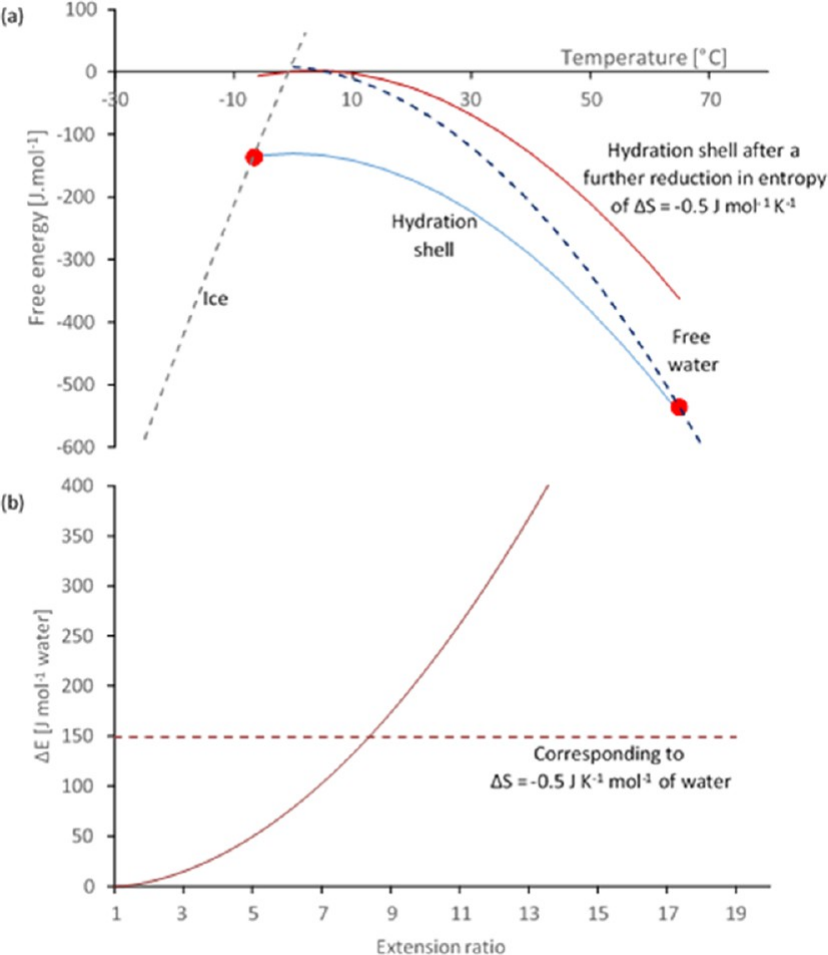
Modeling displacement of the hydration shell through deformation of the fibroin chain (calculations performed using Microsoft Excel). (a) Free energy vs temperature (from data published previously^64^) showing that the hydration shell (blue line) is normally stable, i.e., at lower energy than ice (gray dashed line) or free water (blue dashed line) between the limits marked by red circles but can be destabilized by a further reduction in entropy (red line). (b) Calculation of energy vs extension ratio for fibroin as an entropic spring at 25 °C, assuming active chain segments of 42 kDa (i.e., around nine entanglements per chain^76,79^). The dashed horizontal line in (b) corresponds to the entropy change of Δ*S* = −0.5 J K^–1^ mol^–1^ of water, as modeled in (a).

Then, the question arises, what could cause loss of the hydration shell? One possibility may be that as segments of the protein chain are stretched and oriented by flow, the hydration shell loses more entropy, thereby increasing its energy. A previous work^64^ has already indicated that water in the hydration shell around fibroin has a significantly lower heat capacity (57.4 J K^–1^ mol^–1^) compared with free water (75.3 J K^–1^ mol^–1^ at 25 °C), due to the restricted dynamics of bound molecules, consistent with an entropic penalty relative to free water. Nevertheless, the hydration shell remains at lower chemical potential than free water due to favorable enthalpic interactions (Δ*H* ≈ −525 J mol^–1^ of water at 25 °C). Modeling this (Figure 10a) suggests that the fibroin solution remains stable between the points (marked by red circles) where the plot of the chemical potential of the hydration shell water (in blue) crosses the plots for ice (gray dashed line, around −6 °C) or pure water (dark blue dashed line, around 65 °C). This model also predicts that the hydration shell would become unstable (i.e., at higher chemical potential than free water) following a further relatively small decrease in its entropy. The example shown (solid red line in Figure 10a) is based on a modest additional entropy penalty of Δ*S* = −0.5 J K^–1^ mol^–1^ of water relative to the stable hydration shell around fibroin in NSF prior to gelation.

According to the well-established concept of polymer chains as entropic springs,^57−61^ stretching restricts the number of accessible conformations, producing a decrease in entropy and a corresponding increase in internal energy. The entropy change is often ascribed to a decrease in disorder^137^ as chains are stretched; however, this oversimplification represents a misinterpretation of entropy. Consideration of Boltzmann’s equation^138^
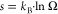
8aor its macroscopic equivalent
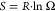
8breveals that Ω is the number of different ways quanta of energy can be distributed among atoms or molecules in different states, and lnΩ is merely a number. This is related to entropy (*s* or *S*) through Boltzmann’s constant (*k*_B_) or its molar equivalent (*R* = *k*_B_·*N*_A_, where *N*_A_ is Avogadro’s number). This returns the emphasis to how energy can be distributed among the available vibrational and translational modes within the system, which also explains the link between entropy change, heat capacity at constant pressure (*C*_p_), and temperature (*T*)
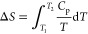
9

For *n* mols of polymer chains undergoing affine deformation at constant volume, the predicted changes in internal energy and entropy are given by^57−61^
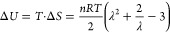
10where λ is the extension ratio (i.e., the length of a stretched chain segment divided by its initial length).

It is assumed that, since it is bound to the fibroin, the hydration shell would experience similar decreases in the numbers of accessible vibrational and translational modes as the protein chain is stretched. On that basis, changes in energy for stretched fibroin at 25 °C were calculated (Figure 10b), which predicted that a threshold of *T*Δ*S* = 149 J mol^–1^ of water (i.e., corresponding to Δ*S* = −0.5 J K^–1^ mol^–1^ of water at 25 °C, as used in Figure 10a) would be exceeded above a stretch ratio of around 8.5. This estimate is somewhat larger than the stretch thresholds to cause changes in chain configurations, as reported by Asakura and co-workers;^126,127^ however, rheological modeling suggested that it could be achieved readily for NSF at modest flow rates, consistent with natural silk spinning, assisted by the increased susceptibility of fibroin to flow-induced chain stretching through the effects of “sticky reptation”.^37^

Although shear flow (rather than purely extensional flow) was used in the present work, it should be noted that this was still expected to produce chain stretching;^37^ the protein chains in NSF are considerably overlapped and entangled; shear rates faster than the slowest relaxation rates were applied and total shear strains in excess of several hundred were applied in the various experiments. Moreover, it may only be necessary to achieve that amount of stretching within a fraction of the fibroin, leading to “gelation nuclei”, which would increase the susceptibility of the remaining protein to flow-induced stretching and account for the gradual gelation observed in this work and previously.^35^

**Figure 11:**
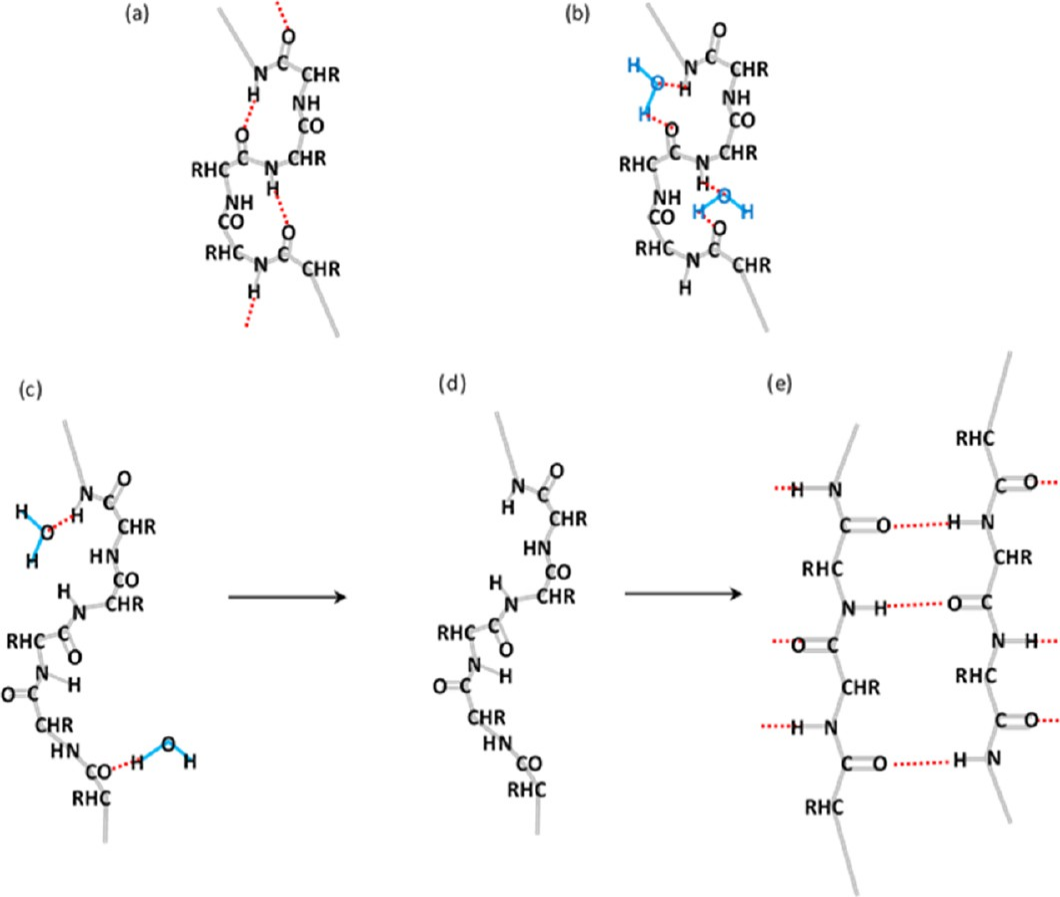
(a) Section of fibroin chain adopting a type II β-turn structure as reported by Asakura et al.^126−132^ with H-bonds (shown in red) between the *n*th and (*n* + 3)th amino acids; (b) proposed structure similar to a type II β-turn but with water molecules (in blue) bridging between the *n*th and (*n* + 3)th peptide groups; and (c) stretched protein chain, with distances between peptide groups too large for bridging water molecules, leading to loss of the hydration shell (d), with the bare protein subsequently forming new peptide-to-peptide H-bonds in β-sheet structures (e), which resists redissolution in water.

A second possibility may be that changes in conformation as the chain is stretched could weaken the bonding structure between the fibroin and its hydration shell. In this respect, several studies by Asakura and co-workers^126−132^ used NMR to measure bond torsion angles along fibroin or model poly(Gly–Ala) peptide chains, suggesting large amounts of type-II β-turn structure in NSF prior to gelation. This structure is characterized by hydrogen bonds between a peptide carbonyl and the N–H group of the amino acid three places further along the chain (Figure 11a). While this may be plausible for protein recovered by gently drying NSF (i.e., silk I), the exclusively peptide-to-peptide H-bonding implied by this structure appears less likely for the protein in aqueous solution, where extensive H-bonding to water molecules is expected.^64^

Instead, we suggest that a slightly modified chain conformation may allow water molecules to bridge between peptide groups (as suggested in Figure 11b). Crucially, these doubly bonded bridging water molecules are likely to be the most strongly bound to the protein, compared with other water molecules attached to the protein by single H-bonds via the peptide hydrogen or oxygen atoms. Moreover, the ability of water molecules to bridge between peptide groups would depend on the (through space) distance between the amino acids. Hence, if chain stretching increased the distance between the amino acids sufficiently (Figure 11c), the H-bonds would weaken, such that water molecules could be released from the protein (Figure 11d), allowing the protein to form new peptide-to-peptide H-bonds (Figure 11e).

Finally, it should also be noted that the two hypotheses presented here are not mutually exclusive. An increase in free energy through an entropy penalty and a loss of H-bonding through conformational changes may operate together, as chains undergo flow-induced stretching.

## Conclusions

The results presented here support the widely held view that natural silk fibers are spun by flow-induced gelation, fueled by a suitably high rate of work input. Birefringence and polarized IR measurements both indicated flow-induced alignment in NSF specimens, which appeared to be a precursor of microphase separation of protein from water. While the rheology of NSF specimens followed the Cox–Merz relationship at low shear rates, separation of water from protein-rich microdomains, leading to shear banding or wall slip, may explain the subsequent deviations in flow curves, producing the peaks and subsequent declines observed in plots of shear stress at higher shear rates.

IR spectroscopy provided further evidence that flow initiated changes in fibroin conformation, with random coil or helical structures in the NSF being replaced by antiparallel β-sheet structure during gelation. Moreover, the spectral changes observed in the O–H stretching region provided a direct experimental observation suggesting water loss from the hydration shell in response to flow, as part of the gelation process. Thus, a link is established between flow-induced gelation of NSF and the thermodynamic behavior reported previously.^64^

## Notes

Institutional Review Board Statement: ethical review and approval were not applicable for this study, which only used invertebrate animals (*B. mori* larvae).

## Notes

Data are available from the authors (PRL) on request, or from the ORDA website at the University of Sheffield: https://orda.shef.ac.uk/.
